# A Solution of the Problem of Population and Subsistence; in a Series of Letters to a Physician

**Published:** 1843-07

**Authors:** 


### ART. XV.

*Solution du Problème de la Population et de la Subsistance, &c.* Par CHARLES LOUDON, Docteur en Médecine, &c.—*Paris*, 1842. 8vo, pp. 336.

*A Solution of the Problem of Population and Subsistence; in a Series of Letters to a Physician.* By CHARLES LOUDON, M.D. &c.—*Paris*, 1842.

IN this publication Dr. Loudon discusses a plan calculated, as he thinks, to prevent population increasing beyond the means of subsistence. It is interesting to the profession, as it professes to be based on certain principles of physiology.

The first four letters are occupied with Malthusian doctrines, and the last two with desultory politico-economical discussions of our poor laws, colonial government, and emigration, Mr. Doubleday's theory of the census, a systematic plan for the division of labour, and the opinion of St. Clement on the exact period of the birth of our Saviour. The remaining letters resolve the problem.

On a previous occasion we observed that the desire of rising in the world, while it checked population, gave rise to the greatest moral evils. The desire to rise does not annihilate the sexual desire; it only substitutes the concubine or prostitute for the chaste wife; and drunkenness, debauchery, and all the crime attendant on loose morals take the place of the quiet virtues of domestic life. Dr. Loudon is of opinion that the number of prostitutes in Great Britain has been much exaggerated; he estimates it at from 60,000 to 70,000, and supposes there is an equal number of females of doubtful character. The mean duration of a prostitute's career is six or seven years; so that the annual demand for virtuous females in Great Britain to recruit the ranks of prostitution is at least 8000 or 9000, or about one every hour. He estimates the annual number of bastards at 40,000; but these are not in general the children of prostitutes, but of virtuous females who have been seduced. As a secondary evil connected with prostitution, Dr. Loudon notices the venereal disease, and quotes the opinions of Richerand and Ricord to the effect that scarcely one per cent. of the men in the middle and higher classes escape infection. Adultery is another evil originating from the moral check alluded to; for men who marry late, take wives much younger than themselves, and hence unhappy matches, since the original disparity in years increases in a geometric ratio as the ages of the parties advance. The sale of young females to old debauchees is another evil cognate with the preceding. Dr. Loudon heard of two instances of such sales in Paris; the prices were £600 and £1000. Other results of this moral check of Malthus are mentioned, and it appears that it is no check at all except upon morals and good order. The licentiousness and debauchery consequent upon the delay of marriage in the higher and middle classes spread with all the rapidity of evil example to the lower, and boys of seventeen have their concubines of sixteen years of age. So far we entirely agree with Dr. Loudon.

Dr. Loudon thinks the laws of nature and of Providence are alike, and that Malthus, not being either a physician or physiologist, could not possibly trace their identity. This our author proposes to do, and so solve his problem. It is a law of nature, according to Dr. Loudon, that man should be monogamous, and that the age of complete puberty ought to mark the period of marriage. This he fixes physiologically at the age of twenty-one years, and shows that marriage at this age has been always accompanied by an improvement in morals. The sexual feelings, however, are strongly developed anteriorly to this period; and to restrain these Dr. Loudon proposes the moral check of affiancing the parties for two or three years previously to marriage; a regular and legal ceremony being performed when the engagement is entered into. We can see nothing objectionable in these plans except their impracticability. It is evident the moral check on marriage at so early an age will still operate; and bachelors still dread the burden of a wife and family, in spite of Dr. Loudon's eloquent arguments in favour of a virtuous union. Our author is prepared for this objection, and insists that if the law of Providence or of nature be followed in one period of procreation, it must be followed in another: and that if it be a law that marriage take place at the age of twenty-one years, it is an equally stringent law that lactation shall be triennial. By this means the number of children will be diminished because pregnancy does not occur during lactation; and in addition, a more healthy state of both mother and offspring will be secured, all the objections to the contrary being set aside; for prolonged suckling neither debilitates the mother nor spoils the beauty of her bust, nor renders the children puny.

These laws being demonstrated, Dr. Loudon calls upon medical practitioners to assist in applying the true, because physiological, check upon population, and so become the greatest benefactors of their species. They are to point out to mothers, as eloquently as possible, the importance and necessity of triennial lactation, and will thus not only do much to prevent prostitution, debauchery, adultery, the sale of virgin chastity, and pinching poverty, in the present generation; but also control that frightful increase of population, which threatens to render it necessary at no far distant period, either to castrate and spay the young folk, or devour the old; a sad dilemma, indeed.

Dr. Loudon will see that we differ from him in his views with respect to the expected increase of population. We think no one measure adequate to control its progress, but we are certain that that general amelioration of man's state termed civilization, and which is the result of numerous concurrent causes, will balance population and subsistence. Independently of these considerations, Dr. Loudon has left out the very key-stone of his plan. He has not proved that lactation, and especially triennial lactation, prevents the recurrence of conception. Dr. Loudon's staple in this matter is not statistics. We have physiological inferences and literary quotations to satiety; but Deville Carystias with his hypothetical sevens is no authority, and the second Book of Maccabees is at least physiologically apocryphal. We would even venture to question whether the opinions of "Nurse" in Romeo and Juliet are to be considered of weight, although Dr. Dickson would think them conclusive. Dr. Loudon refers to the only statistical inquiries within his reach, and these are certainly opposed to his views; we refer to the inquiries of Mr. Roberton of Manchester, who found that fifty per cent. of the females belonging to the labouring classes in that town became pregnant during lactation. Dr. Loudon explains away this opposing statement very ingeniously, by observing that the females of the class alluded to are away from their children during the whole day, and only return at night to find them asleep. In fact, they do not properly, that is to say, physiologically, suckle their children; consequently, conception may readily take place. In natural lactation, according to Dr. Loudon, the infant ought to have the breast every two or three hours. There lately appeared in the Dublin Medical Press, some statistical inquiries made by Dr. Laycock of York on this subject, which corroborate Mr. Roberton's deductions, although the individuals of whom the inquiries were made were taken from different classes of society. It appears that 209 conceptions took place during 766 lactations, 27 per cent.; and that of 135 married females, 76 or 56 per cent., became pregnant while suckling, according to the highest estimate, and 33·9 per cent. according to the lowest. We refer to the paper itself. That there is some antagonism between the mammæ and ovaria or uterus is probable; if, however, this be fully conceded, it is obvious that the balance between these organs is readily disturbed in favour of the uterus or ovaria, whenever anything occurs to interrupt lactation even in a slight degree. These interruptions are of necessity continually occurring in civilized life, especially among the labouring classes, and rendering the law inoperative. What then becomes of Dr. Loudon's plan to affiance at an early age and marry at twenty-one?

Having differed so much from Dr. Loudon's views we must do him the justice to say, that this attempt to elucidate political economy by the laws of physiology, and to improve the morals of the people through medical doctrines and precepts, is exceedingly meritorious, and we cordially wish Dr. Loudon success in similar future undertakings. The style of the work exhibits haste and carelessness; but Dr. Loudon appears to have been limited to time by a wish to get out his publication previously to the discussion of the poor-laws during last session.

